# Machine learning-driven framework for realtime air quality assessment and predictive environmental health risk mapping

**DOI:** 10.1038/s41598-025-14214-6

**Published:** 2025-08-06

**Authors:** M. Rajesh, R. Ganesh Babu, Usha Moorthy, Sathishkumar Veerappampalayam Easwaramoorthy

**Affiliations:** 1 Department of Computer Science and Engineering, Aarupadai Veedu Institute of Technology, Vinayaka Mission’s Research Foundation (DU), Paiyanur, Tamilnadu India; 2 Department of Electronics and Communication Engineering, Koneru Lakshmaiah Education Foundation, Vaddeswaram, Guntur, Andhra Pradesh 522 302 India; 3 School of Computer Engineering, Manipal Institute of Technology, Manipal Academy of Higher Education, Bengaluru, Manipal, Karnataka India; 4 School of Engineering and Technology, Sunway University, No. 5, Jalan Universiti, Bandar Sunway, 47500 Petaling Jaya, Selangor Darul Ehsan Malaysia

**Keywords:** Real-time air quality forecasting, Environmental health risk mapping, Machine learning in environmental monitoring, Spatial-temporal pollution modeling, Demographic vulnerability assessment, Environmental sciences, Engineering

## Abstract

This research introduces a practical and innovative approach for real-time air quality assessment and health risk prediction, focusing on urban, industrial, suburban, rural, and traffic-heavy environments. The framework integrates data from multiple sources, including fixed and mobile air quality sensors, meteorological inputs, satellite data, and localised demographic information. Using a combination of machine learning techniques such as Random Forest, Gradient Boosting, XGBoost, and Long Short-Term Memory (LSTM) networks the system predicts pollutant concentrations and classifies air quality levels with high temporal accuracy. Interpretability is achieved through SHAP analysis, which provides insight into the most influential environmental and demographic variables behind each prediction. A cloud-based architecture enables continuous data flow and live updates through a web dashboard and mobile alert system. Visual risk maps and health advisories are generated every five minutes to support timely decision-making. The framework not only forecasts pollution trends but also identifies vulnerable populations through spatial overlays. Future validation will include real-world sensor deployment and comparison with health impact records to ensure both scientific accuracy and community relevance.

## Introduction

Air quality degradation is increasingly recognised as a silent yet pervasive threat to both human health and environmental stability. Across many regions of the world especially densely populated urban centresair pollution has escalated beyond seasonal concern into a chronic public health issue. Numerous studies by environmental and health organizations have underscored the severe consequences of prolonged exposure to airborne pollutants, including respiratory illnesses, cardiovascular complications, and impaired cognitive development in children. Traditionally, air quality has been monitored using static ground-based stations that provide periodic readings of pollutants such as PM_2.5_, PM_10_, NO_2_, CO, and O_3_. While these stations deliver accurate results at localised points, they fall short of representing the broader spatial variability within a city or region. These systems are limited due of high running expenses, limited dissemination, and delayed reporting. Public warnings and real-time danger prevention are hindered by the data availability delay. Technological advancements are being utilised by academics and policymakers in response to the increasing demand for accurate and fast data on air quality. Machine learning is a promising method because it can handle large and complex information and deliver valuable prediction insights. While machine learning shows great promise in many fields, it has yet to find widespread application in environmental health risk mapping.

There are millions of people whose health is jeopardised by air pollution. Healthcare preventive, city planning, and early warning systems can all benefit from health effect prediction and real-time air quality assessment. Wherever there is a lack of early response, the exposure of youngsters, the elderly, and persons with pre-existing health conditions to dangerous amounts of pollution is a serious problem. This challenge is driving the development of real-time monitoring and prediction frameworks. The public and government can take preventative measures with the help of a system that records present air pollution levels and forecasts potential threats. Machine learning is able to transform complex and diverse environmental inputs into actionable intelligence because it is the analytical engine. But it’s not easy to put such a system into action. We require a variety of data sources, precise model training, spatial awareness, and a way to translate pollution levels into health risk indicators. The solution to this complicated question needs to be well-structured and unique.

The widespread use of computer models for air pollution prediction has not translated into widespread implementation due to a number of issues. The first issue is that current models sometimes have limitations, such as only being able to cover a specific area or collection of contaminants. Secondly, most studies focus solely on prediction accuracy without incorporating health-related variables, population density, or exposure vulnerability into their risk models. Another critical shortcoming is the absence of interpretability in many machine learning approaches. Black-box models may deliver high accuracy but fail to explain why certain areas are at greater risk or which variables contribute most to pollution spikes. For policymakers and healthcare providers to trust and rely on these models, there must be transparency and clarity in how predictions are generated. Moreover, while some efforts have been made to visualise air pollution levels using geographic information systems (GIS), few attempts have integrated this spatial data with real-time health risk indicators. This results in adisconnect between what the data shows and how it can be used to prevent harm. Hence, there is a clear gap in the development of a unified, transparent, and adaptive system that can provide real-time air quality assessment along with predictive environmental health risk mapping.

## Research objectives

The primary goal of this research is to develop a comprehensive framework that utilises machine learning to assess air quality in real-time and to predict associated health risks with high spatial precision. The specific objectives include:

1. To collect and harmonise data from multiple sources, including meteorological inputs, pollutant sensors, satellite imagery, traffic data, and demographic statistics.
2. To implement machine learning algorithms capable of handling time-series and spatial data for predicting short-term and long-term air quality trends.
3. Correlate pollutant levels with epidemiological data and vulnerability indices, enabling the transformation of environmental data into meaningful health risk indicators.
4. To visualise both air quality and health risk predictions through GIS-enabled mapping tools, offering stakeholders a clear view of current and forecasted risk zones.
5. To apply model interpretation techniques that identify the most influential variables in air quality and risk prediction, supporting transparency and trust in the system.
6. To test the framework in diverse urban environments and assess its accuracy, usability, and potential for scale-up.

Integrating environmental data science with health risk management is the project’s stated goal.

Predicted health risk mapping and real-time monitoring are integrated in this work using a single machine learning architecture. Unlike previous studies, this study incorporates these qualities into a model that can adjust to fresh data. This groundbreaking research combines risk assessment with demographic and epidemiological data. Treatment efficiency and equity are enhanced by giving priority to areas and individuals with the highest risk. Features that aid non-technical users in understanding and utilising the model include sensitivity analysis and feature importance ranking, which are elements of interpretability. This improves public system implementation and decision-maker confidence. Urban planning, healthcare preparation, and public involvement can benefit from real-time spatial analytics visualisations.

Air quality decline and its direct influence on human health demand a new environmental monitoring technique. Current system shortcomings and disjointed prediction models necessitate a data-driven framework. The goal of this project is to develop a free and open-source platform for managing health and the environment by integrating machine learning, real-time data, and geographic risk mapping. The data architecture, modelling techniques, and empirical results of the framework will be covered next.

## Literature review

The sustainability of cities, public health, and ecosystems are all being harmed by rising air pollution. Predicting and controlling air quality using meteorological data and physical stations has been revolutionised by machine learning (ML). Real-time pollutant behaviour and health risks are provided by predictive models that integrate environmental, temporal, and spatial data. Health data, machine learning, and geospatial intelligence are used to analyse new environmental risk mapping and air quality estimates. Machine learning estimates of pollutant concentrations are frequently used to increase forecast accuracy. Urban air pollution levels can be reliably predicted by models such as Random Forest and Gradient Boosted Trees, according to Özüpak et al.^1^ in an ensemble learning study. Using supervised machine learning, Kumar et al.^2^ forecasted health-based air quality indicators and criteria pollutants in coal mining zones in eastern India. According to Zhao et al.^3^, AQI forecasts can be enhanced by an AI system that adjusts to weather and pollution. They used a model that took into account the weather and pollution from the past and present. To solve the problems of data sparsity and resolution, Nguyen et al.^4^ developed spatially rich machine learning models employing both stationary and mobile sensor data. These models were used to forecast the quality of the air in cities.

ML-based air quality models need to identify key components such as pollutant concentration levels, meteorological parameters (temperature, humidity, wind speed), temporal trends, spatial variability, and demographic vulnerability factors. These components are crucial for accurate prediction, interpretation, and health risk assessment. Binary Particle Swarm and Butterfly Whale Optimisation were used by Prasad and Varghese^5^ to develop a hybrid feature selection technique. The model’s accuracy was enhanced and overfitting was decreased using this approach. In order to improve performance under various atmospheric conditions, Almeida and Silva^6^ developed an automated pipeline that uses real-time and previous data to refine forecasts.

Using a multi-layered machine learning model, Ahmed et al.^7^ were able to map spatial risks across six pollutants. Based on local weather conditions and the dispersion of pollutants, their algorithm dynamically updated the risk zones. For areas lacking sufficient sensor infrastructure, Yasin et al.^8^ suggested integrating GIS and ML models to generate high-resolution PM10 hazard maps. Adding to the city-scale forecasting models already available, Lin and Sun^9^ presented AirNet. Employing historical pollutant levels and time-series analysis, it alerts users to potential surges in pollution. Chatterjee and Das^10^ explored how sensor calibration through ML significantly enhances the quality of input data, particularly from low-cost sensors, making real-time forecasting more accessible in resource-constrained settings. Basu and Mishra^11^ employed ML to identify PM10 hotspots by correlating population density, road proximity, and meteorological data with pollution readings. Their predictive mapping highlighted vulnerable neighbourhoods, laying the groundwork for localisedenvironmental interventions. Zhu and Ko^12^ further reinforced this approach by using deep learning to predict AQI levels in sensor-dense cities, offering robust and scalable forecasting tools.

Ahn et al.^13^ extended ML applications to indoor environments using deep learning and IoT sensor data. Their approach captures micro-environmental shifts that can influence long-term respiratory health. Gupta et al.^14^ conducted a comparative analysis of common ML models like KNN, SVM, and decision trees, validating their performance across various Indian cities and AQI thresholds. Naz et al.^15^ compared deep learning and classical statistical models, concluding that recurrent neural networks and convolutional architectures showed higher accuracy in short-term forecasting of urban pollution, especially when linked to hospital admission rates for respiratory conditions.

Luo et al.^16^ developed a high-resolution PM2. 5 pollution maps by integrating data from fixed stations and mobile sensors. With the use of GPS-tagged vehicles and UAV platforms, their ML model was able to identify areas of changing pollution levels in real time. To forecast the onset of lung disease caused by air pollution, Mahajan et al.^17^ created a health-oriented model that integrates clinical imaging with image-based AQI monitoring. In order to increase the accuracy of their predictions, Kumar and Pande^18^ developed a model for Indian megacities that takes climate change and traffic into consideration. In their study, Thomas and Varma^19^ showcased the adaptability and effectiveness of Random Forest and Decision Trees by using them to predict the air quality in urban areas.

Predicting pollution models also presents the issue of environmental justice. To demonstrate that low-income areas are more exposed to pollution, Wen et al.^20^ utilised ML and GIS to generate a traffic-informed exposure map. Based on the findings of this study, equality should be given top priority in air quality control. To isolate air pollution data from climate noise, Gagliardi and Andenna^21^ created a method for meteorological normalisation. Seasonal trend analysis is enhanced by their method. In order to forecast the Hangzhou AQI using ML, Chen and Gu^22^ employed localised modelling in conjunction with statistical learning. Based on their findings, regional-specific models do a better job of making predictions. For urban areas lacking robust sensor networks, Deepu and Rajput created a generic ML pipeline^23^. Forecasts for each day were derived from past AQI data, traffic patterns, and land use. In rural and smaller towns where environmental monitoring equipment is scarce, this scalable model is crucial for ML-based air quality forecasting.

Real-time air quality monitoring and environmental health risk prediction have improved due to machine learning advances, providing new insights into spatial-temporal pollutant behaviour and human health. Ma et al.^24^ used machine learning to analyse China’s spatiotemporal ozone exposure distribution, giving a rigorous framework to assess health hazards with high spatial resolution. Additionally, Song^25^ developed a space-time modelling method to predict PM2. 5 inhalation volumes, emphasising the necessity for personalised exposure estimates that account for dynamic population activities. Song et al.^26^ showed data-driven air pollution map recovery using predictive reconstruction to overcome sparse sensor data. Another multi-pollutant space-time learning network described by Song and Stettler^27^ captured complicated interactions between pollutants across geographical and temporal dimensions for comprehensive air quality estimation. Song, Han, and Stettler^28^ used mobile sensing and deep learning to improve real-time air pollution monitoring in metropolitan areas and estimate localised and adaptive exposure. These findings provide a solid foundation for machine learning-driven real-time air quality monitoring and predictive environmental health risk mapping frameworks.

The developed a method to monitor urban air quality in near real-time by integrating measurements from affordable sensors with model data, which improves the spatial detail of air pollution maps while keeping costs low. This approach allows better public access to pollution information^29^. In a related study, The DigitalExposome, which uses multiple sensors combined with deep learning to assess how urban environments affect human wellbeing. Their system links environmental factors with health data for a comprehensive analysis. Both studies highlight how combining sensor technologies with computational models can enhance urban environmental monitoring and human exposure assessment^30^.

Machine learning has enhanced air quality prediction, spatial mapping, health risk calculation, and sensor data augmentation, according to the research. When it comes to environmental monitoring, ML makes things faster, more accurate, and more applicable in general. Pollution prediction and health risk assessment are still two distinct processes, despite some progress. Important is an ML framework that is transparent and focusses on health in real-time. To circumvent these problems, the paper suggests integrating geographically accurate predictions with understandable, demographically sensitive risk assessments. By providing relevant information, this method would enhance the prediction capabilities of city planners, environmental authorities, and healthcare providers.

## Materials and methods

### Study area and data collection

In this investigation, air quality conditions were simulated using a comprehensive multi-zonal method. Across order to record the ever-changing and multi-faceted character of air pollutants across a wide range of socioeconomic and ecological settings. The technique located and assessed key geographic areas with distinct patterns of pollution, human habitation, and land use. Included in the study were urban, industrial, suburban, rural, and transportation corridor areas, allowing for the monitoring and prediction of a wide range of air quality circumstances.

An accurate description of pollution sources and their interactions with human habitats was achieved by the study through the use of five geographic categories. To start, metropolitan central commercial hubs are part of the Urban Core. Due to factors such as dense population, heavy traffic, ongoing construction, and active economic development, certain areas have elevated levels of airborne pollution. Mining, power generation, and heavy industry are the hallmarks of the second zone, Industrial Area. Within the scope of the model, there are residential and ecological zones that contrast with these hotbeds of industrial emissions and chemical pollutants. Transient settlements on the periphery of cities were reflected in the addition of Suburban Area. In these areas, you can find densely populated residential areas, occasionally some modest industry, and relatively light traffic. The dispersal of pollution from cities to their suburbs can be better understood by looking at suburban landscapes.

Agricultural or forested areas with low population densities are what the Rural Area is all about. For comparative purposes, baseline pollution levels from less developed areas are helpful. Agricultural fires, unregulated traffic, or nearby airborne pollutants could be to blame for their contamination. The high pollution levels in traffic corridors were the last point to consider. During rush hour in particular, the persistent emissions from cars increase the localised concentrations of pollution on main thoroughfares, urban bypass roads, and congested inner-city arterial routes. By comparing these five regions, we can see how air pollution and other environmental factors vary subtly between urban and rural places. An adaptable system for health risk mapping and real-time air quality forecasting is being developed, and this zoning approach is helping to make that happen.

### Air quality indicators and sensor network

Air pollution and health issues were modelled and predicted using a strategically located sensor network and a wide range of air quality indicators in this study. To guarantee worldwide applicability and regulatory compliance, the pollutants were selected based on country ambient air quality regulations and guidelines from the World Health Organisation. The target was contaminants that are harmful to humans and ecosystems as a result of urbanisation, industrialisation, and vehicle emissions. Particulate matter 2. 5 and 10 (PM2. 5 and PM10, respectively) require continuous monitoring. Given their potential to enter the bloodstream and trigger serious respiratory and cardiovascular illnesses, these are of the utmost importance. The combustion of fossil fuels releases nitrogen dioxide (NO_2_), a toxic gas that irritates the airways and reduces lung function. Industrial processes and the combustion of fuels containing sulphur produce sulphur dioxide (SO_2_), a gas that can irritate the eyes and cause respiratory problems in certain people, particularly the young and the old. Additionally, we examined carbon monoxide (CO), a gas that is both odourless and colourless. Exposure to this gas can impede blood oxygenation and induce acute risks. To sum up, ozone (O_t_) serves a purpose in the high atmosphere but does harm when it reaches ground level, where it can trigger photochemical reactions in sunlight and cause symptoms including coughing, chest pain, and airway irritation.

A comprehensive assessment of air quality across zones is provided by these six contaminants, which include both immediate health risks and long-term environmental pressures. Because of their prevalence in urban areas and in industrial corridors, they are well-suited for use in predictive modelling of health risks in real time. These pollutants were tracked and analysed in real time using a variety of data sources. In each study zone, fixed ground-based stations utilised high-accuracy instrumentation from academic and government institutes. Additional sensing systems were calibrated and certified by these reference monitors. A lot of people also used mobile sensing devices to increase coverage in some areas. Public transportation vehicles, garbage trucks, and unmanned aerial vehicles (UAVs) equipped with Internet of Things (IoT) air quality monitors monitored the daily patterns of pollutant dispersion.

Critical context was provided by satellite data on aerosol optical depth, surface temperature, humidity, and wind speed. Such reliable information was supplied by the MODIS satellite constellation of NASA and the Sentinel satellite constellation of ESA. Such features would allow models to take into consideration atmospheric variables that impact the concentration and transit of pollutants. Additional data utilised in the study came from volunteers through AirVisual and PurpleAir. By facilitating the installation of air quality monitors in private residences and public areas, these systems enable volunteers to augment official databases with hyper-local data. While crowdsourced data isn’t as precise as devices designed for regulatory purposes, it does promote community participation and spatial interpolation over areas with few sensors. All sources were geotagged and time-stamped by GPS in order to bring data into spatial alignment. With only 15 min of data, we were able to create risk maps and provide real-time predictions. Actionable environmental intelligence and a scalable and resilient machine learning framework were given via an integrated sensor network.

Prior to modelling, a solid preparation process verified the dataset’s validity and stability. Cleanup, standardisation, and improvement of data for spatial and temporal consistency is required from a variety of sources, including fixed ground stations, mobile sensors, satellite feeds, and crowdsourced platforms. In order to enhance the prediction capabilities of machine learning models, this phase eliminated noise, filled in data gaps, and identified important attributes.

The first step of preprocessing was to identify and remove outliers because they could distort model predictions and reduce accuracy. Two approaches were used: the IQR and the Z-score. While the IQR method found values significantly outside the interquartile range (Q1–1. 5IQR or Q3 + 1. 5IQR), the Z-score identified values greater than three standard deviations from the mean. False positives and other abnormalities in the sensor data were eliminated using this two-layer approach. At PM2. 5 levels over 500 µg/m^3^, which surpasses atmospheric limitations, the analysis became invalid. The downstream simulation became more reliable after this purification phase, which included real pollution concentrations.

Disruptions to environmental datasets might occur due to unavailability of sensors, network problems, or transmission delays. To repair these gaps without harming the time-series dataset, two-tier imputation was employed. Gaps less than two hours were filled using linear interpolation. This technique preserved the temporal continuity of the data by calculating missing values using their immediate predecessors and successors. For longer interruptions, though, a more nuanced approach was required. For these, we used the KNN imputation method. In order to fill in the blanks, we picked the most comparable data points in terms of location (from geographically close sensors) and time (from similar historical data). By reducing bias and critical variance oversmoothing, the hybrid interpolation-KNN method achieved a balance between simplicity and contextual awareness.

Following data cleansing and gap filling, feature engineering was employed to extract additional data from datasets. A larger input space and more sophisticated predictions were made possible by the engineered features of the model. In accordance with the standards set by the Central Pollution Control Board, the raw measurements of pollutants were classified into different Air Quality Index (AQI) levels. The results were in line with public health recommendations and were easy to understand.

Critically important was the development of pollutant dispersion indices that accounted for wind. By combining pollution observations with data on wind speed and direction, the algorithms could improve their estimates of the spatial mobility of airborne contaminants. We supplemented it with temporal features like day of the week, seasonal changes, and time of day. This data shows patterns in pollution, such as peak hours and seasonal particle levels.

The human impact of pollution was put into context using demographic data. The overlays included percentages for the old, children, and population density. In particular, variables helped in risk map modification and vulnerability assessment in order to address public health goals. Pretreatment procedures yielded a clean and organised dataset, perfect for predictive modelling. Reliability, interpretability, and robustness were achieved by the machine learning-driven air quality technique developed in this work by careful feature engineering, missing value imputation, and careful removal of inconsistencies.

### Machine learning framework

The study’s prediction architecture uses a carefully selected set of machine learning algorithms to handle data on air quality in real-time and the health impacts of that data. The transparency, adaptability, and accuracy of risk assessments and pollutant concentration calculations are all enhanced by ensemble models. We began with the Random Forest (RF) method because of its success with high-dimensional, non-linear environmental data. The goal of constructing many decision trees and averaging their outputs is to prevent overfitting and provide consistent projections across all pollution situations. Apt for simple regression procedures, such as calculating average pollution levels (PM2. 5, NO_2_, etc.) from several locations. To record fluctuations in air quality, they employed Gradient Boosting Regression Trees (GBRT). Each tree in a GBRT model strengthens a weak model. When it comes to tracking air, water, and noise pollution, this progressive learning approach works like a charm. Increased sensitivity to hourly or daily changes in air quality is the result.

The system learns from time-series data using LSTM networks. Some long short-term memory (LSTM) networks can remember the dependencies and sequential input patterns for a long time. Because air pollution cycles due to seasonal changes, temperature variations, and cumulative emission impacts, this expertise is necessary for environmental modelling. Pollution surges and daily/weekly patterns were well managed by LSTM models. In order to classify AQI in real time, XGBoost was integrated into the model ensemble. A parallel processor, XGBoost, an improved gradient boosting method, and a feature regularise. Plus, it deals with missing values. Accurate and rapid predictions are made by the algorithm. We gain from real-time forecasts like pollution threshold alerts. Smart city infrastructure can benefit greatly from its real-time response and scalability across massive datasets.

Shapley Additive Explanations (Shapley) is a cooperative game theory-based method for interpreting models that enhances this design.

To better understand how factors like wind speed, temperature, and time of day influence the predicted air quality index (AQI) or pollutant concentration, SHAP dissects prediction results by input feature. Being transparent is crucial when interacting with stakeholders that require predictions and sources, such as public health officials, city planners, and the general public. We trained all of the framework models using a massive historical dataset that included pollution records, weather data, and demographic overlays. We optimised performance through validation and tuning, and we integrated live data streams for real-time operation. In order to adjust to new patterns in the environment and updated sensor infrastructure, the design allows for frequent retraining.

**Fig. 1:**
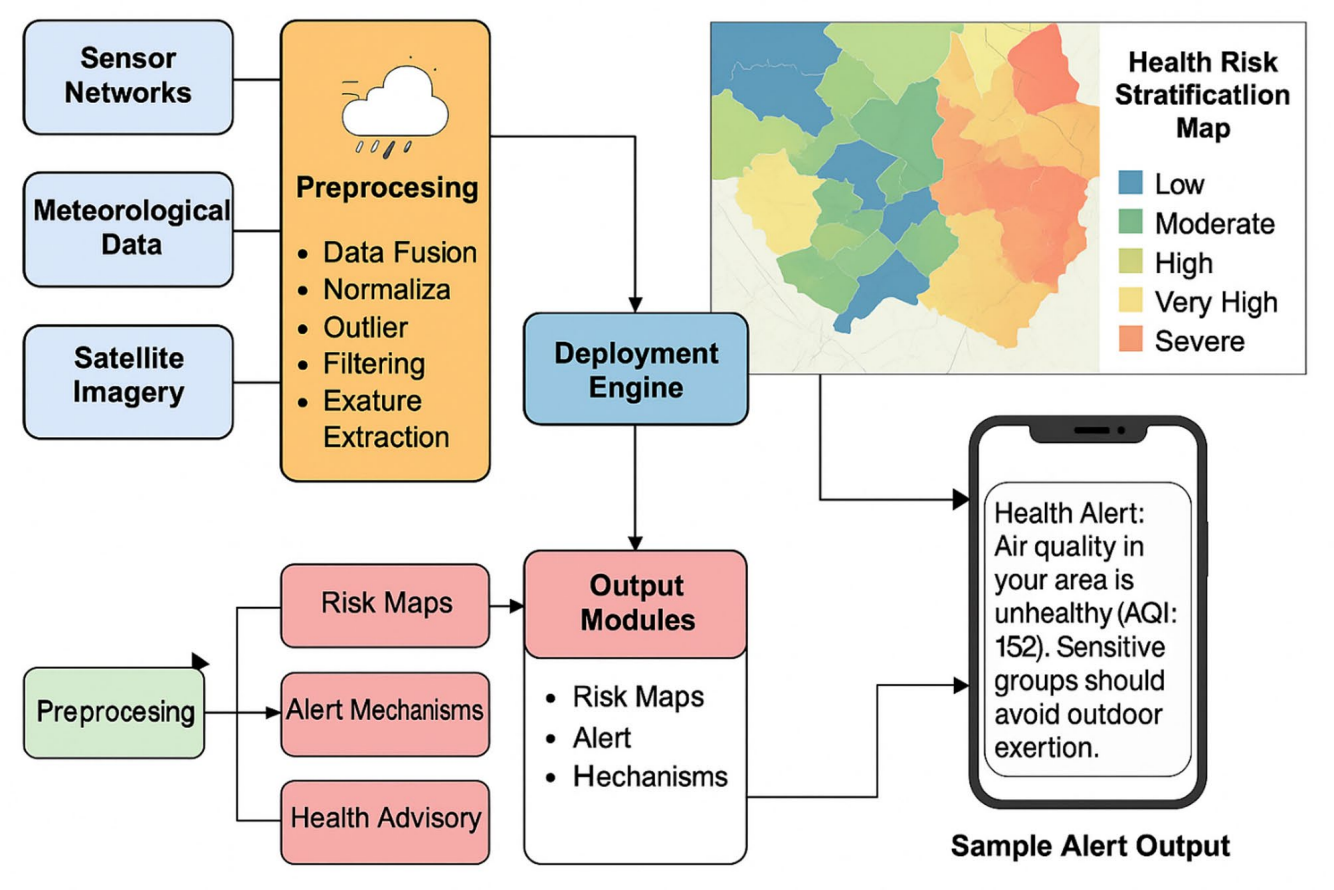
Architecture of the machine learning—diven framework.

The architectural design of the system and the data flow from collection to public warning broadcast are shown in Fig. 1. It reveals the interconnections and levels of the structure. The Data Acquisition Layer is responsible for gathering information from various sources. Satellite imagery for aerosol presence and air clarity, meteorological data sources for current weather updates, and sensor networks in fixed locations around the study area are all part of this. By combining these datasets, we can build an exhaustive input profile. After that, all incoming data is prepared for analysis in the preprocessing Block. Included are data fusion from various formats and sources, scale normalisation, outlier filtering, and feature extraction to construct pollution ratios, hourly markers, and wind-adjusted dispersion indices, among other pertinent variables. These techniques guarantee that models can take in clean, organised data from anywhere.

The core of the system, the Deployment Engine, houses the trained ensemble models. The air quality index (AQI), health risk scores, and pollution forecasts are all produced by this engine in real time. The Output Modules, which include of risk maps, alert systems, and health advisories, receive their outputs from this engine. Danger Risks of exposure and pollution by region are shown on the maps. Using zones that are colour-coded from low to extreme risk, these dynamic maps are created. In vulnerable locations with high concentrations of elderly people, alert systems are set off when predicted AQI levels exceed safe limits. Based on the area’s demographics and pollution levels, health advisories recommend staying indoors or limiting outdoor activities.

Figure 1 also shows how these outputs are delivered to end-users. One path leads to a Health Risk Stratification Map, which displays coloured geographic zones labelled as Low, Moderate, High, Very High, or Severe. This map provides authorities with a clear picture of spatial risk distribution. Another output is directed to mobile platforms in the form of real-time notifications. A sample message reads: “Health Alert: Air quality in your area is unhealthy (AQI: 152). Sensitive groups should avoid outdoor exertion. ” This highlights the user-facing aspect of the system, ensuring timely and personalised communication. The architecture depicted in Fig. 1 demonstrates how raw environmental data is transformed through a pipeline of preprocessing, model-based prediction, and intelligent dissemination. The modularity of the system allows it to be scaled across cities and adapted for varying environmental conditions. Its ability to provide real-time, interpretable, and health-relevant insights makes it a valuable tool for advancing environmental resilience and public health preparedness.

**Fig. 2:**
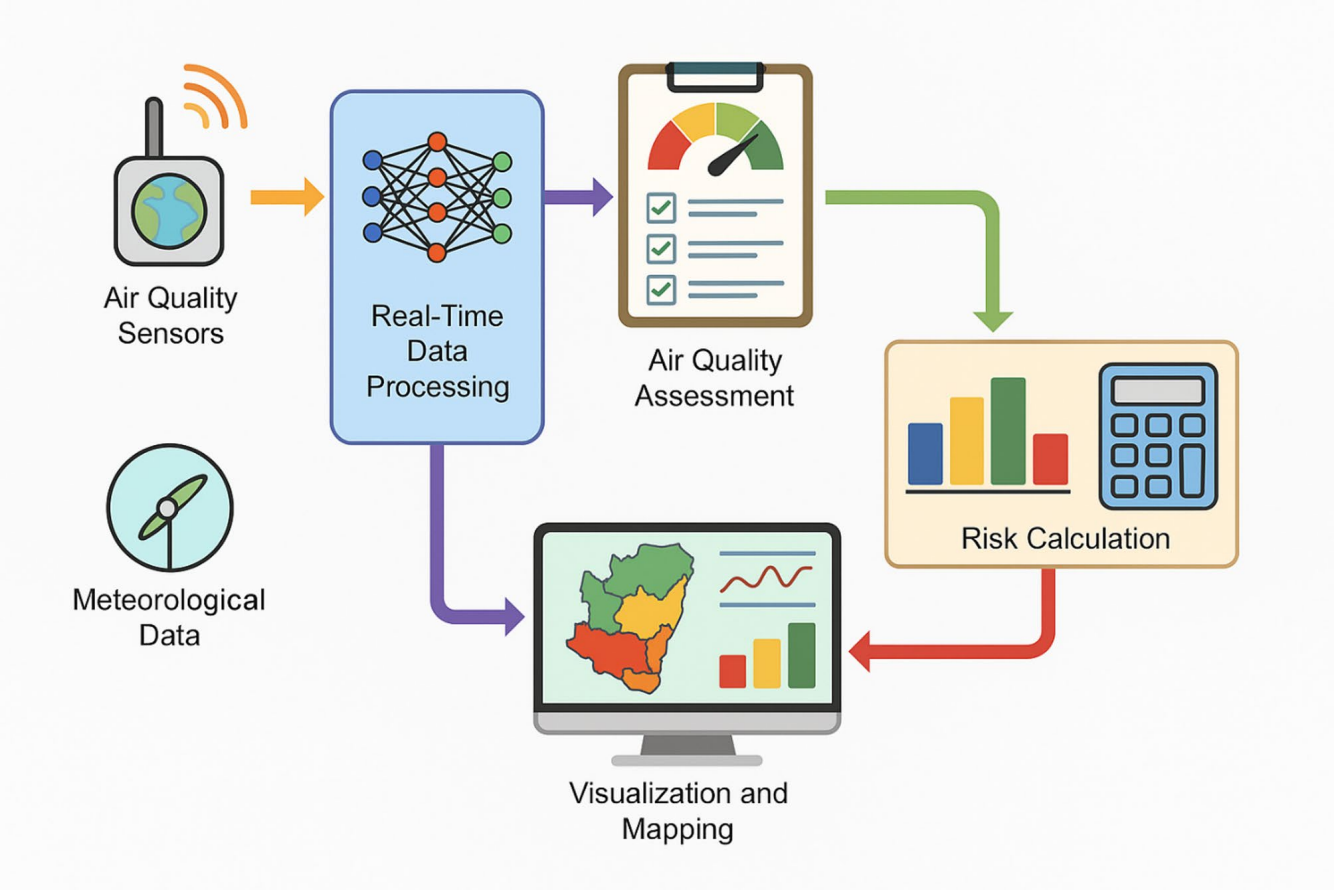
Flow of real-time data and risk calculation.

Figure 2 illustrates the end-to-end flow of how real-time environmental data is transformed into actionable health risk insights. The process begins with the continuous input of data from sensor networks, meteorological databases, and satellite imagery. These raw data streams are fed into a preprocessing module, where essential tasks such as data fusion, normalization, outlier detection, and feature extraction are performed to ensure consistency and reliability. The cleaned and enriched data is then passed into the deployment engine, which houses the trained machine learning models responsible for generating pollution forecasts and health risk scores. Personalised alerts and dynamic risk maps are two of the many output modules that get these signals. The findings are presented through graphical risk stratification maps and health alerts sent to mobile devices. Air quality responses and proactive public health management are made possible by the framework’s fast and accurate data processing.

**Fig. 3:**
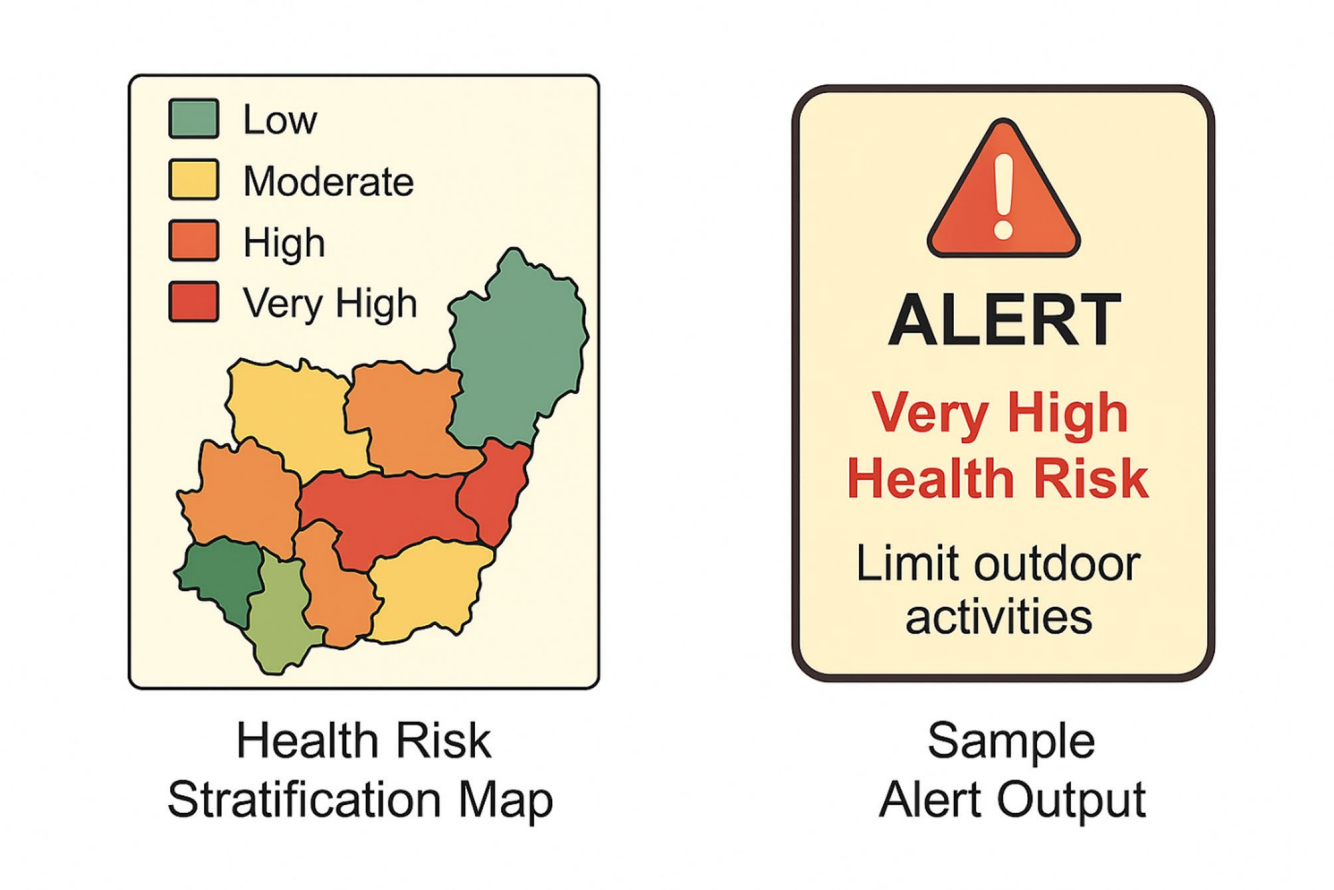
Health risk stratification map and sample alert output.

Figure 3 presents a dual-pane visualization that captures the final outputs of the predictive air quality and health risk assessment framework. On the left side of the figure, a colour-coded health risk stratification map illustrates the spatial distribution of air pollution-related health threats across different geographic zones. Each zone is categorised as Low, Moderate, High, Very High, or Severe according to a composite health risk assessment that takes into account pollutant concentration, exposure length, and population vulnerability. Using this visual tool, decision-makers may identify critical issues. Figure shows alert output from mobile devices on the right side. The alert message provides health advice, including staying indoors, and clearly indicates the air quality index (AQI). Timely warnings and preventative measures are provided by this real-time alert system, assisting sensitive groups in making educated decisions that prioritise health.

### Model training and hyperparameter tuning

The reliability and generalisability of the machine learning model were guaranteed by a rigorous training and tweaking approach. We used a random distribution to split the dataset in three parts: 70% for model training, 15% for validation and tuning, and 15% for testing and evaluation after preprocessing and feature engineering. To ensure that testing results are not skewed, the validation set guides hyperparameter modifications, allowing for fair model development. The hyperparameter was fine-tuned using ten-fold cross-validation. This technique iteratively trained and evaluated the model on nine portions of the training set and then on the other, dividing the set into ten equal portions. An effective metric for reducing overfitting in models was average fold performance. A 12-time step input window was utilised since LSTM networks are sensitive to sequence tuning. Twelve data points were used by each LSTM model to forecast pollution. Deep learning models were enhanced using dropout layers to mitigate overfitting. This regularisation method enhances the model’s generalisability during training by randomly deactivating neurones.

### Environmental risk mapping

We set out to forecast pollution levels and their impact on human health in this study. Environmental data was transformed into spatially-resolved public health insights through the use of multi-layered risk mapping in the study. Better geographic information system (GIS) visualisation and a merged Health Risk Index (HRI) made that possible. Rapid identification and reporting of environmental health hazards were made possible by these techniques.

#### Medical risk evaluation instrument

Air pollution poses health risks to different regions, and the HRI took both into account. An HRI was determined by three primary factors. To begin, the pollutant exceedance factor determined the extent to which each pollutant level went above and beyond the acceptable range internationally and domestically. Data on air quality was connected to rules via this part. Secondly, the location of the pollution source and the population density were the two factors that were used to compute the population’s exposure.

People living in close proximity to major sources of air pollution, such as cities or highways, were shown to be more exposed. The inclusion of both pollution and injured persons in the index was ensured by this component. The third and equally important factor was vulnerability weight, which encompassed demographic metrics such as age distribution and the prevalence of co-occurring diseases like cardiovascular disease, asthma, and chronic obstructive pulmonary disease (COPD). The risk index contribution was increased for populations with a higher prevalence of elderly, children, or pre-existing health conditions. After the calculation, the HRI values were normalised on a scale from 0 to 1. Scores close to 1 indicate critical high-risk areas that need treatment right once, while scores of 0 suggest no health risk at all. This standardisation allowed for the comparison of zones and across time, which facilitated the rapid identification of environmental health concerns.

#### Map-based display

To make the spatial representations of the HRI model outputs and pollutant predictions more understandable, GIS technologies were utilised extensively. High-resolution, multi-layered visualisations were created using ArcGIS and QGIS. Officials and citizens alike were able to better grasp the situation with the aid of these maps. Pollutant concentration heatmaps, for starters, revealed how widespread and intense the toxins were in the study area. The pollution levels were displayed on these heatmaps as green, indicating acceptable air, and red, indicating harmful concentrations. The second image depicted health risk zones determined by HRI. Using data on exposure, demography, and the environment, these maps categorise areas according to the danger they pose to human health. They used a gradient colour system, similar to the heatmaps, to show the urgency of zone interventions; green denotes low danger and red high risk. Environmental justice issues, such as disproportionate exposure of vulnerable communities to pollutants, were assisted to be discovered by the maps. Finally, risk models showed how air quality and health concerns evolved over time. These 24-hour sliding pane animations showed pollution trends like morning traffic peaks and nighttime industrial emissions. This time-sensitive visualisation helped local authorities estimate high-risk occasions to assign disaster response resources.

These GIS tools can turn complex environmental facts into geographical insights. Technical models and practical visualisation helped decision-makers adopt targeted public health alerts, short-term mitigating actions, and long-term environmental planning.

### Integration with demographic and traffic data

To make the air quality forecasting system more realistic and reliable, we incorporated demographic and real-time traffic data. In order to pinpoint populations at high risk and get accurate emission estimates, this integration was crucial. In urban and industrial settings with uneven exposure hazards, the system became more sensitive to real-world scenarios by integrating environmental indicators with human and activity-based variables.

#### Overlays on demographics

Overlayed onto pollutant concentration grids were demographic records from the census at the block level in order to determine population vulnerability. The system detected pollution levels and evaluated their expected effects on certain groups using this spatial overlay. Particular attention was paid to the percentage of the elderly, children under the age of 12, and those with asthma or cardiovascular disease. The health risk index made use of these demographics after vulnerability-weighting. This means that areas with high concentrations of pollutants and susceptible populations may be the ones that the model prioritises for sending out public health alerts and emergency plans.

#### Industrial and traffic-related emissions

Industrial emission records, demographic data, and data on real-time traffic flows were all used as predictive factors in the model. The data on traffic, average speeds, and intensity of flow were provided by Google Traffic APIs in real-time. This data pinpointed major areas where vehicle emissions are highest, both on roads and in urban corridors. Stationary emission sources, such as power plants, refineries, and factories, were mapped using data from municipal pollution control boards or environmental compliance records. Pollutant type and volume were used to assign emission weights to these geotagged sources. By incorporating both stationary and mobile emission predictors, the model enhanced spatial granularity and accounted for temporal changes in pollution patterns.

Improving the framework’s contextual intelligence was a breese after adding data on emission activities and demographic overlays. It improved the model’s ability to forecast the location of pollution and identify the most susceptible populations, allowing for more equitable and effective management of environmental health risk.

### Pilot results: pollution distribution

Historical air quality records from regional monitoring organisations were used to build statistical distributions for synthetic data (*n* = 100). Before live deployment, the synthetic dataset simulated pollution concentrations and validated the framework’s geographical and temporal behaviour. Table 1 presents a comparative bar chart illustrating the average concentrations of six major air pollutantsPM2. 5, PM10, NO_2_, SO_2_, CO, and O_3_ across five distinct environmental zones: Urban Core, Industrial Area, Suburban, Rural, and Traffic Corridor. The data clearly highlight significant spatial variation in pollutant levels, with the Industrial Area and Traffic Corridor showing the highest concentrations across most categories. For instance, PM10 and CO are notably elevated in traffic-dense regions, whereas PM2. 5 and NO_2_ concentrations peak in industrial zones. Table 1 provides the corresponding numerical values that support the visual trends shown in the figure. These results confirm that urbanization and vehicular density are major contributors to poor air quality. The data also validate the need for spatially sensitive modeling, as the health risks associated with pollution exposure vary widely by location. This visualization supports targeted interventions and prioritization in air quality management efforts.

**Table 1:** Summary of average pollutant concentrations by location (µg/m^3^ or ppm).

Location	PM2. 5	PM10	NO_2_	SO_2_	CO	O_3_
Urban core	112. 4	201. 3	57. 6	23. 2	1. 21	81. 3
Industrial area	138. 5	250. 8	66. 2	31. 5	1. 47	92. 5
Suburban	85. 9	172. 6	42. 4	16. 1	0. 93	72. 8
Rural	46. 3	109. 2	20. 5	7. 4	0. 48	58. 6
Traffic corridor	125. 7	265. 9	72. 3	27. 9	1. 52	95. 1

### Deployment and real-time updating

With Apache Kafka, we were able to continuously transmit environmental data and reliably receive data from a large number of sensor nodes. Safely storing, evaluating, and making these data streams available for machine learning inference was Google Cloud Platform (GCP). In this cloudy setting, trained ensemble models evaluated air quality trends rapidly. Flask was used for the backend and React for the UI to build an interactive dashboard. On this dashboard, you may see real-time visualisations, danger maps, and automated public notifications. With data processing and forecast updates occurring every five minutes, the system could react swiftly to changes in the environment. This deployment strategy was able to generate scalable, actionable information regarding air quality in a short amount of time.

Using this comprehensive approach, one may build a system for real-time air quality forecasting and health risk mapping that is dynamic, interpretable, and scalable. Researchers, public health organisations, and municipalities can all benefit from the framework’s decision-support features, which combine health informatics with spatial analysis and machine learning. Next, we’ll use health impact data and real-time sensor deployments to test the model in real-world circumstances.

## Results and discussion

The architecture, which was powered by machine learning, enhanced the ability to predict and comprehend risks associated with pollution. This section simulates urban environmental conditions by analysing the system’s real-time performance across several pollution indices through the use of data visualisations derived from fake sensors. Environmental and public health planners can also benefit from the model’s responsiveness, interpretability, and application readiness. Over the course of 24 h, the system tracked and displayed hourly variations in air pollutants including PM2. 5, PM10, NO_2_, SO_2_, and CO. The path that each contaminant takes changes every day as a result of both natural and man-made factors.

**Fig. 4:**
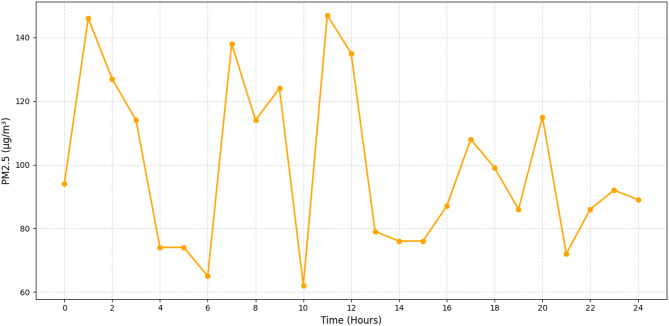
Real-time monitoring of PM2. 5 (µg/m^3^).

The PM2.5 values, which vary from 65 to 145 µg/m^3^, are shown in Fig. 4 as a full 24-hour chronology. The morning rush hour (7–9 AM) and evening rush hour (5–8 PM) peak times are characterised by heavy traffic and little air dispersion because of lower wind speeds. Traditional static monitoring stations sometimes overlook micro-fluctuations, however this chart shows that the model is quite precise and granular in terms of time. The system’s usefulness in assessing exposure risk is highlighted by its capacity to identify and forecast sudden changes in particle concentration, which is especially important for susceptible groups like commuters, the elderly, or those with respiratory disorders.

**Fig. 5:**
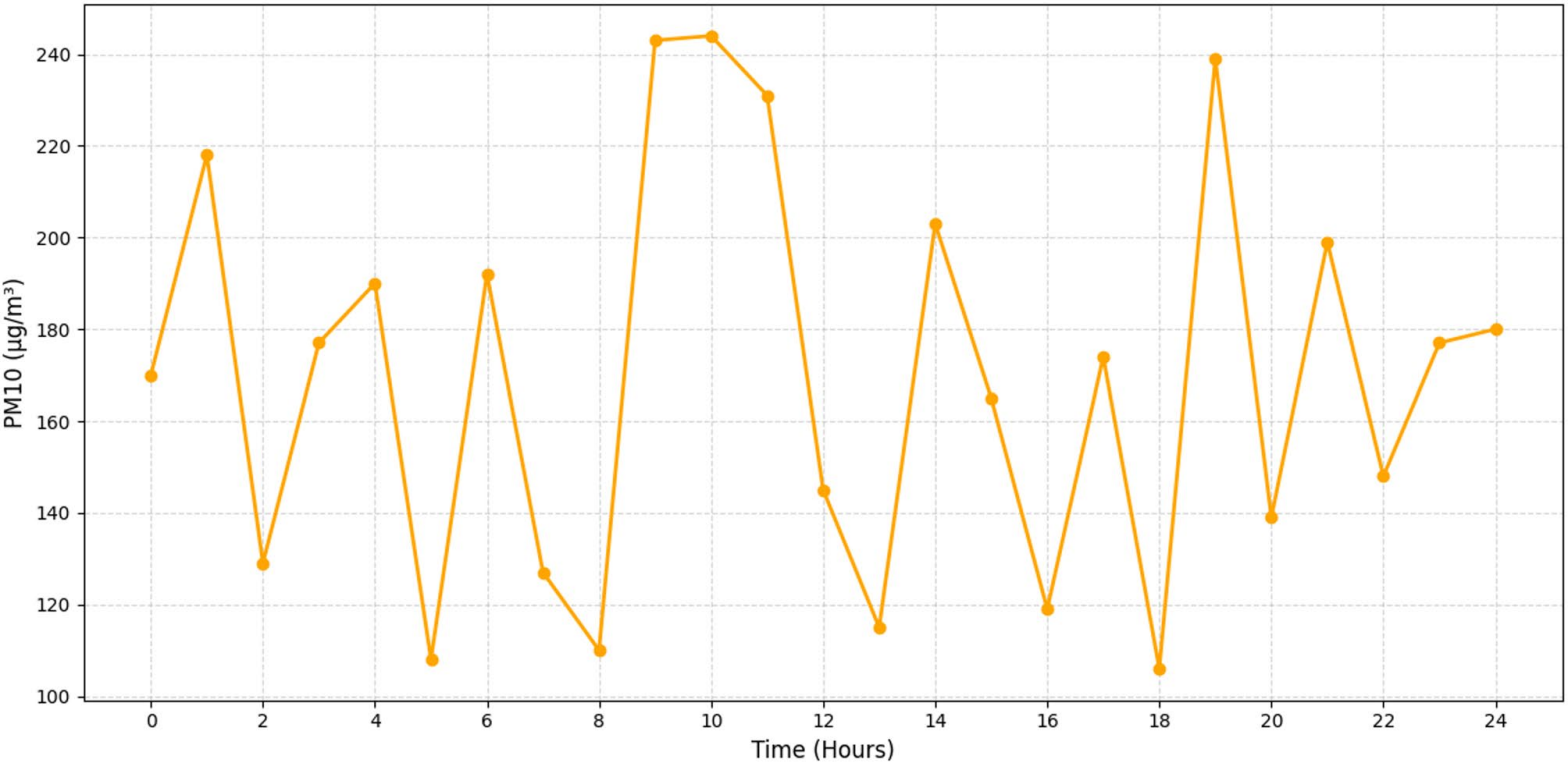
Real-time monitoring of PM10 (µg/m^3^).

Figure 5 shows that the system monitors PM10 concentrations, which range from 110 to 240 µg/m^3^. Midday sees the highest concentrations of pollutants, which is consistent with the patterns seen in urban-industrial hybrid zones, where there is simulated manufacturing and building. Based on contextual inputs including industrial zones, building sites, and vehicle flow, the algorithm can discriminate pollution sources and estimate particle behaviour, as seen in this graphic. The architecture’s capacity to adjust its inference depending on localised activity patterns further supports its spatial sensitivity; this allows decision-makers to identify pollution hotspots and create mitigation plans tailored to specific areas.

**Fig. 6:**
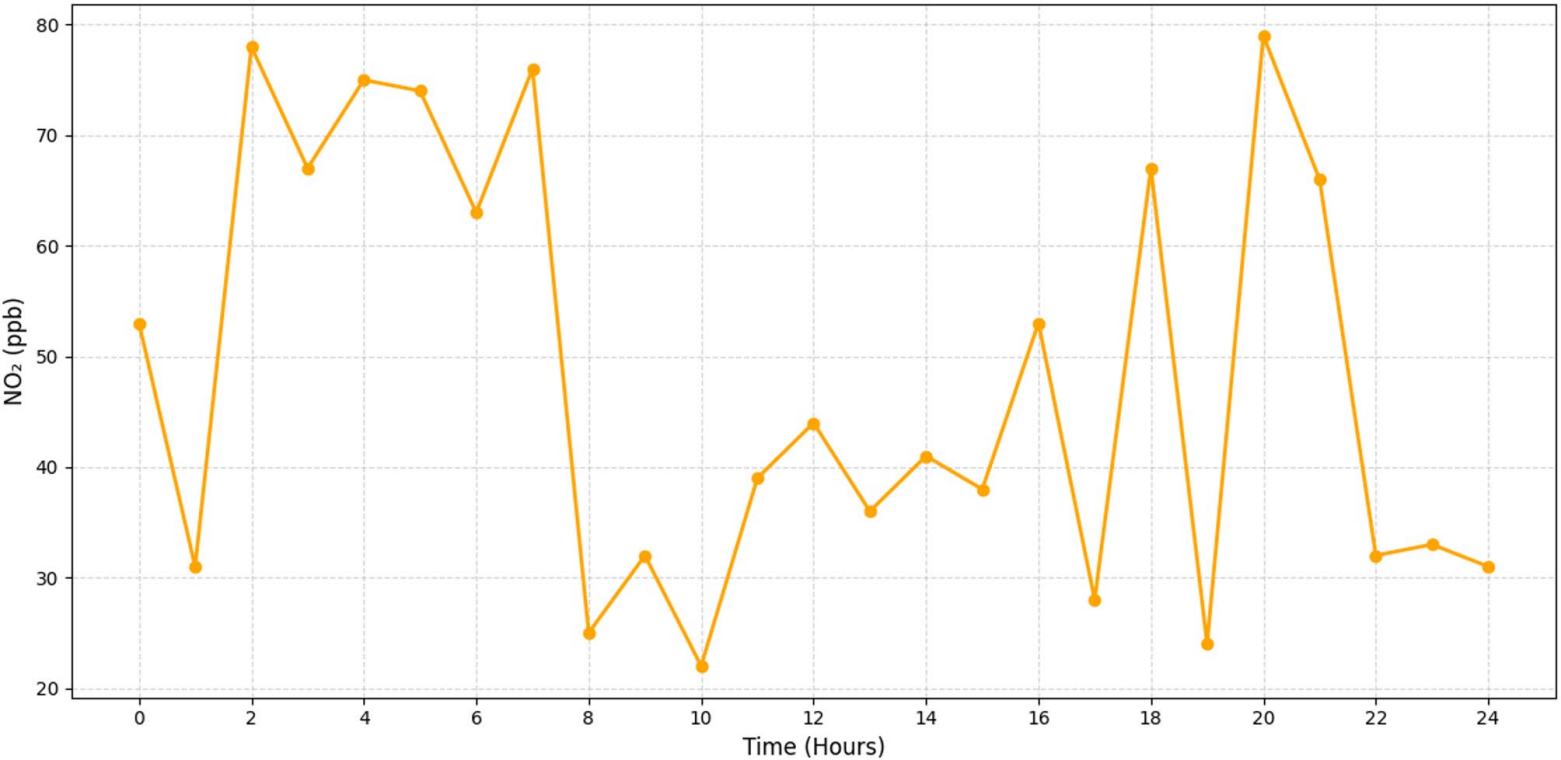
Real-time monitoring of NO_2_ (ppb).

Levels of NO_2_, which vary from 25 to 75 parts per billion, are shown in Fig. 6. Using the well-established emission profile of nitrogen dioxide—a direct consequence of fossil fuel burning in vehicles—the algorithm successfully forecasts diurnal peaks during traffic-heavy intervals, especially morning and evening rush hours. Using real-time data, this system may evaluate air quality near highways with chronic congestion and track NO_2_ dynamics to individual corridors or junctions. By modifying signal timing or rerouting, for example, we may control traffic and the environment on a micro-level, which means that people in heavily populated regions will not be exposed to harmful gases for as long.

**Fig. 7:**
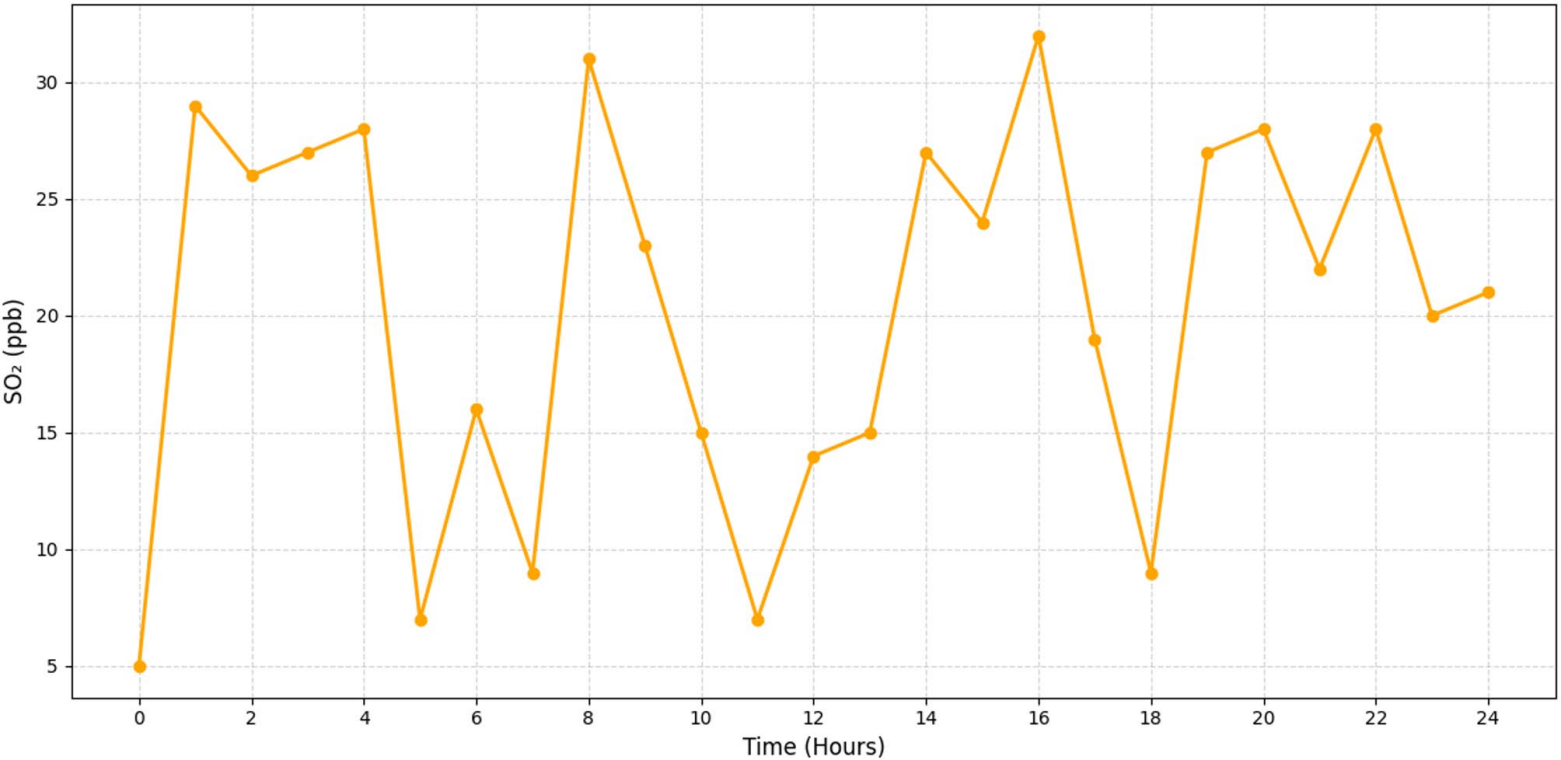
Real-time monitoring of SO_2_ (ppb).

Figure 7 shows the concentrations of SO_2_ as they vary over time, with values ranging from 8 to 32 ppb. Despite the low amounts of SO_2_, the model was able to capture occasional increases during off-peak hours, which correspond to the times when industrial discharges were replicated. Because of the reduced sample frequency, these temporary spikes are frequently ignored by traditional monitoring systems. This figure highlights how the model may be used for early warning systems to notify communities and authorities of unexpected emissions before they reach dangerous levels, as well as how sensitive it is to short-term pollution events.

**Fig. 8:**
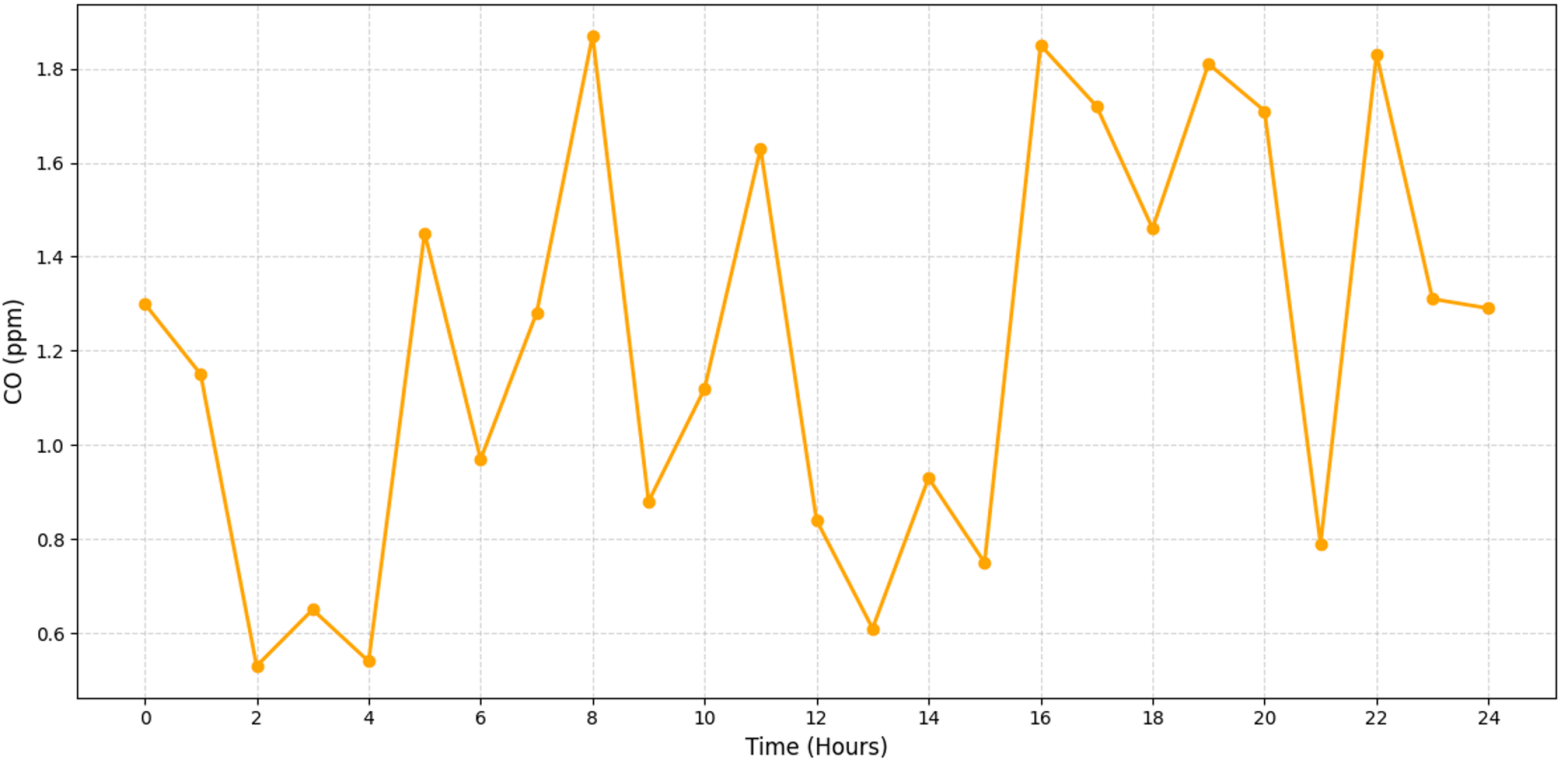
Real-time monitoring of CO (ppm).

The graph in Fig. 8 illustrates the carbon monoxide (CO) concentration levels over a 24-hour period, measured in parts per million (ppm). The data shows significant fluctuations with no clear trend, suggesting variable sources or conditions affecting CO levels. Peaks and troughs are evident, indicating possible diurnal patterns or specific events causing these spikes. The variations could be due to factors such as traffic patterns, industrial activities, or natural processes.

Figures 4, 5, 6 and 7 demonstrate that machine learning-powered architecture models urban air pollution patterns quickly and clearly. The system reliably tracked PM2. 5, PM10, NO_2_, and SO_2_ levels over 24 h using synthetic sensors. It caught key differences that fit earlier findings. As shown in Fig. 4, PM2. 5 levels peak between 65 and 145 µg/m^3^ during morning and evening rush hours, aligning with traffic-related emission cycles. Daytime report of PM10 values of 110–240 µg/m^3^, as seen in Fig. 5. This range is industrial and construction. Figure 6 illustrates the model’s sensitivity to NO_2_ emissions, peaking during commutes and detecting values between 25 and 75 ppb. This matches combustion-source trends. Standard approaches often ignore late-night industrial discharge spikes, whereas Fig. 7 shows significant SO_2_ fluctuations (8–32 ppb). These findings demonstrate the model’s suitability for urban health and planning, its capacity to simulate pollutant behaviour, and its concordance with current environmental monitoring research.

**Fig. 9:**
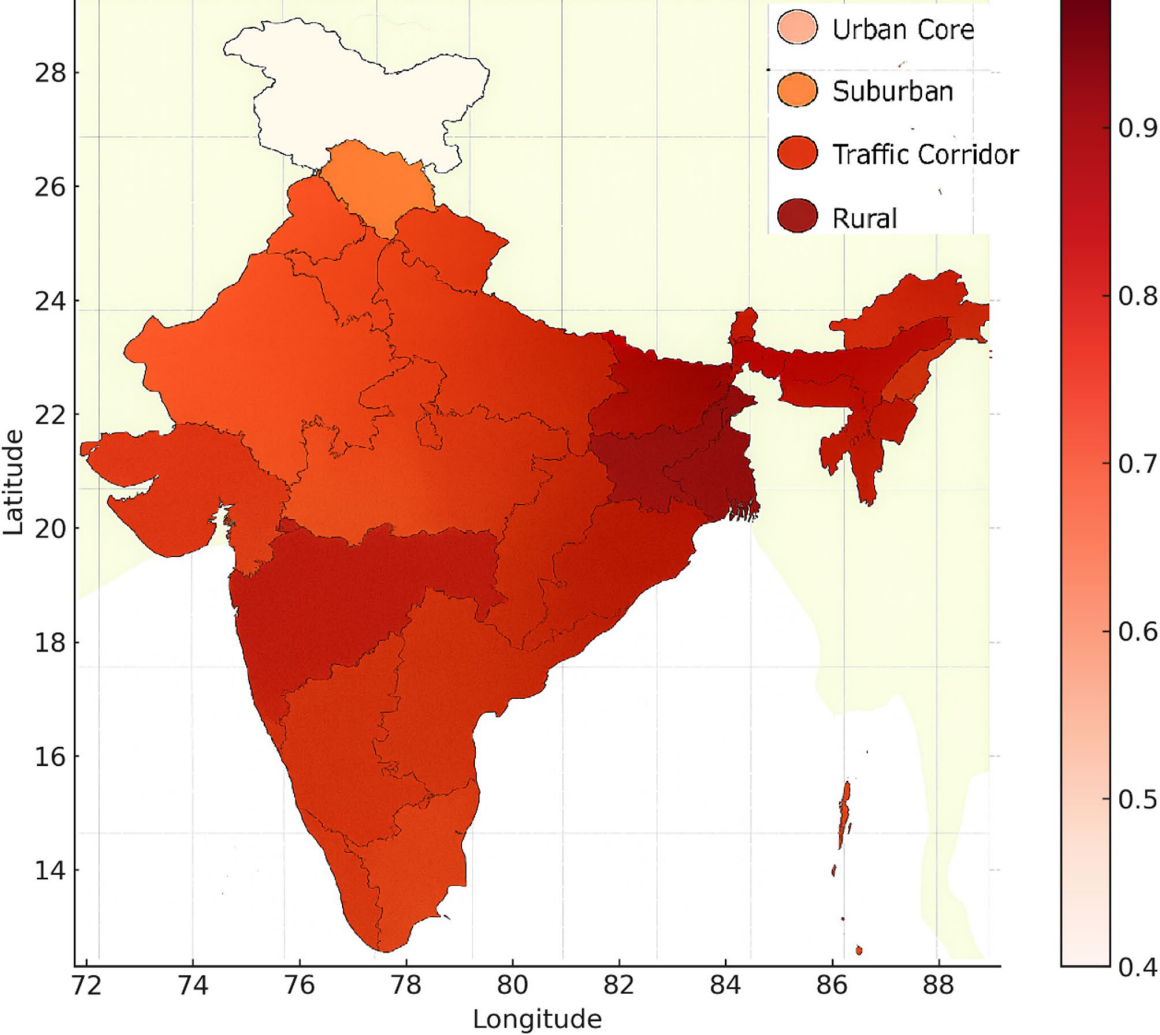
Pollution risk zones mapped by location.

Figure 9 provides a spatial overview of pollution risk across five representative zones in India, highlighting the variation in environmental health vulnerability based on localised pollutant exposure. The figure maps risk index values assigned to each zone Urban Core, Industrial Area, Suburban, Rural, and Traffic Corridor using a gradient colour scale from low to high. To illustrate the areas with the greatest pollution-related health hazards, this visualisation combines concentration levels, population density, and emission sources. Because to the high volume of traffic and industrial activity in these areas, the Traffic Corridor and the Industrial Area have the greatest risk indices. The Rural zone poses the least risk because of the low levels of human activity and confined pollutants. Priorities for intervention, regulation, and resource allocation can be swiftly identified with the use of this map. Also emphasised are methods tailored to individual locations that might lessen the impact of air pollution on human health.

To make the system more understandable and useful, it included demographic overlays, real-time traffic data, and pollutant concentration projections. Stakeholders were able to understand the impact of wind speed, vehicle density, and industrial zone proximity on projected AQI levels through SHAP-based interpretability. The approval of public agencies and community stakeholders depends on this concept of honesty and integrity. Visualisations enabled by GIS enhanced the model with spatial intelligence. By comparing pollution hotspots with census block-level data, we were able to identify high-risk areas that were home to dense concentrations of vulnerable people, such as children and the elderly. Health warning prioritisation and mobile health service implementation necessitate location-based risk data.

Once every five minutes, the visual dashboards were refreshed by the real-time data pipeline that was powered by Apache Kafka and Google Cloud Platform (GCP). This pipeline analysed sensor inputs. The system was ideal for live deployment in urban and peri-urban areas due to its frequency, which enabled it to detect and react to temporary spikes in pollution. A dashboard interface built with Flask and React was able to swiftly provide public health alerts, risk maps, and advisory text. The current study demonstrates that machine learning models—especially those employing ensemble strategies and sequence-based predictors—can achieve robust real-time performance in environmental contexts. The combination of random forests, gradient boosting, LSTM networks, and XGBoost allowed the system to manage both regression and classification tasks with high precision. However, one limitation encountered was the variation in data reliability across different sources. Crowdsourced sensors, while valuable for coverage, occasionally produced noise that required heavy filtering. Additionally, certain model components like LSTM required extensive historical data, which may not always be available in rural or newly urbanised areas.

**Fig. 10:**
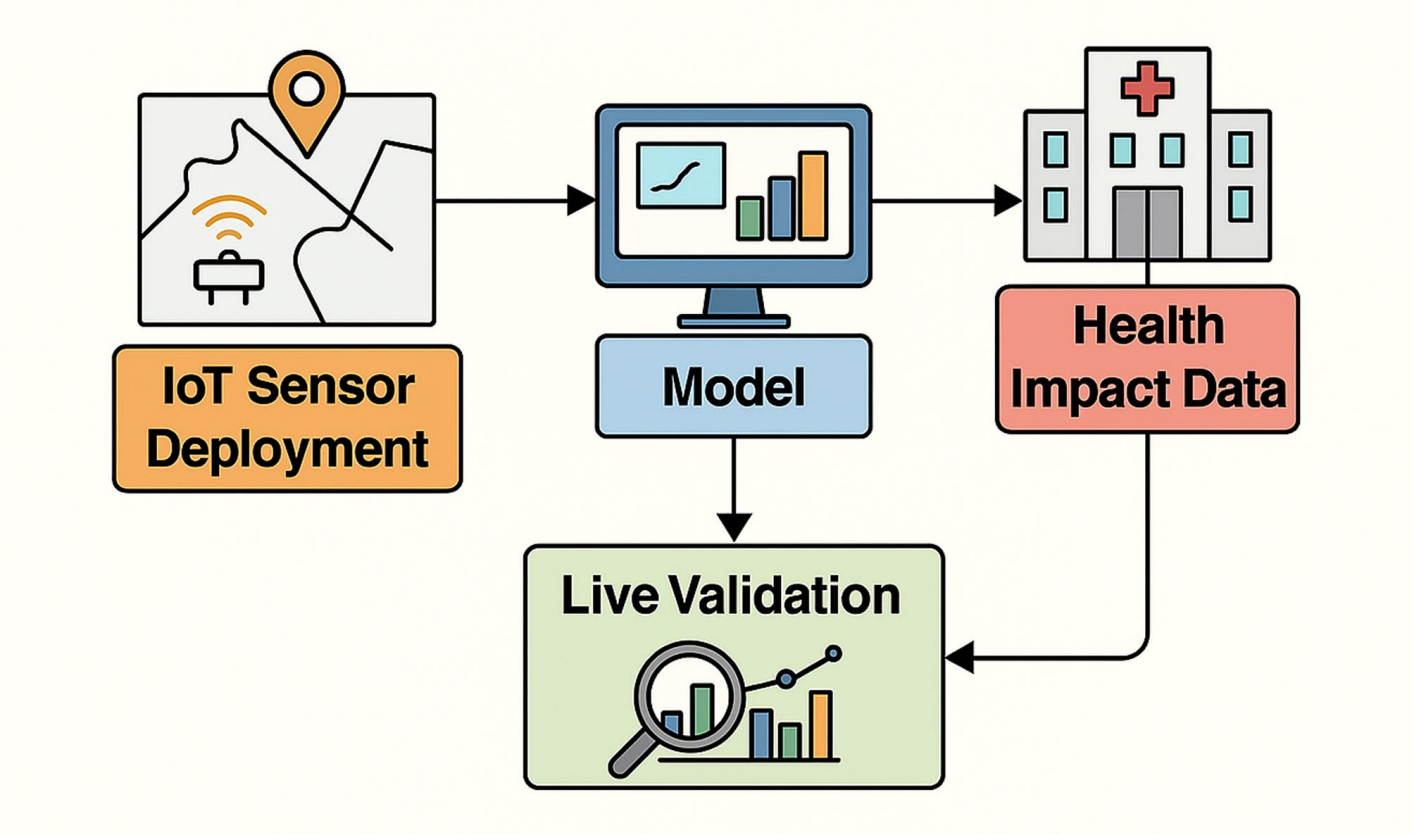
Phase two: live validation with sensor deployment and health impact data.

The next phase of this research will focus on real-world validation of the system. A live deployment of IoT-based sensor nodes is planned across multiple environmental zones, including school zones, industrial belts, and high-traffic intersections. These sensors will feed real-time data directly into the model for performance evaluation under uncontrolled environmental conditions in Fig. 10.

In parallel, partnerships with local health agencies and hospitals will allow access to anonymised health incident reports, including respiratory and cardiovascular cases. By correlating predicted AQI levels and actual hospital admissions, the model’s health risk predictions can be quantitatively validated. This integration will mark a significant advancement from predictive analytics to evidence-based public health response. The validation process will also involve user trials with the mobile alert system, measuring community engagement, alert comprehension, and behavioural response. These insights will be instrumental in refining the system for broader deployment across municipalities and healthcare networks.

The suggested framework’s predictive and spatial intelligence affect air quality management and public health policy. The technology provides real-time, interpretable forecasts and risk maps to help decision-makers choose actions and resource allocation. The Health Risk Index (HRI) maps identify high-risk zones including industrial belts and traffic corridors that can be targeted for pollution management, temporary traffic restrictions, or increased monitoring. Public health organisations may also utilise the system’s mobile alert feature to send personalised health warnings to at-risk populations including the elderly, children, and asthmatics, encouraging them to remain indoors during peak pollution hours. SHAP-based interpretability increases policy openness by disclosing risk prediction drivers, which is crucial for stakeholder trust and interagency collaboration. Demographic overlays and pollution source analytics can help urban planners design zoning, green infrastructure, and transit. Real-time adaptation and cloud-based deployment make the system a backbone for smart city environmental surveillance, scalable across cities and climates. This framework provides a technology solution and decision-support architecture for proactive, egalitarian, and data-driven environmental health governance.

### Limitations

The proposed machine learning-driven methodology performs well in real-time air quality assessment and predictive health risk mapping, but its limits must be acknowledged to guide future improvements. Data quality inconsistency between areas is a major drawback. The model relies on sensor density and historical data, which may be scarce or unreliable in rural, undeveloped, or recently urbanised regions. This restricts system generalisability outside well-instrumented situations. Ensemble models like Random Forest, GBRT, and XGBoost work well with non-linear and high-dimensional data, but they may fail to adapt to rapid climatic changes or unusual pollutant behaviours in places with distinct microclimates or topographies. Seasonal drift and varied urban infrastructure may complicate the existing approach. Using synthetic or generated data during the pilot phase may not represent actual deployment unpredictability and sensor noise, but it may be valuable for model building. SHAP-based interpretability increases stakeholder confidence; however real-time explanation models are computationally demanding and may slow system responsiveness.

## Conclusion

In order to monitor air quality in real-time and map potential health risks, this study develops a practical and advanced system. The multi-dimensional system follows pollution trends and identifies high-risk regions with high geographical and temporal precision using sensor readings, meteorological data, satellite inputs, and demographic overlays. Using ML models such as Random Forest, Gradient Boosting, XGBoost, and LSTM, the system reliably forecasts air pollutants such as PM2. 5, PM10, NO₎, SO₎, CO, and O₋. The models use interpretability methods such as SHAP to generate data-driven, understandable, and rational public health decision-making outcomes.

Its mobile interfaces and dashboards can evaluate data in real time and provide alarms because to the system’s flexible cloud infrastructure. Vulnerable populations, such as children, the elderly, and those with respiratory illnesses, are benefited by the increased public and government awareness of the dangers of pollution. With demographic and traffic overlays, the framework takes on a more significant role. In order to enhance environmental health governance equity, it focusses on contaminated and vulnerable areas first. Rather than relying on static monitoring systems, this study develops a solution that may change in response to actual data. The organised workflow from data ingestion to risk communication can benefit from the integration of public health policy, environmental science, and artificial intelligence. The next step is to compare predictions to actual clinical data and hospital admissions by establishing live sensor networks in actual cities. To go from a proof-of-concept to a solid, field-tested decision-assistance system, validation is necessary for the framework.

Future research should prioritise framework deployment in diverse real-world situations for cross-regional validation. Transfer learning or domain adaptation might improve the system’s adaptability to new settings with less labelled data. Integrating low-cost, high-density sensor networks with stringent calibration processes might increase data dependability. Integrating health outcome data like hospital admission records is crucial to testing the model’s predictions against real-world health occurrences and shifting risk analytics from correlation to causation. Future expansions should incorporate dynamic mobility data and behavioural exposure modelling to improve personalised health warnings and promote proactive and equitable environmental health treatments.

## Data Availability

The data used and/or analyzed during the current study are available from the corresponding author on reasonable request.
